# The Critical Role of Codon Composition on the Translation Efficiency Robustness of the Hepatitis A Virus Capsid

**DOI:** 10.1093/gbe/evz146

**Published:** 2019-07-10

**Authors:** Lucía D’Andrea, Francisco-Javier Pérez-Rodríguez, Montserrat de Castellarnau, Susana Guix, Enric Ribes, Josep Quer, Josep Gregori, Albert Bosch, Rosa M Pintó

**Affiliations:** 1 Enteric Virus Laboratory, Department of Genetics, Microbiology and Statistics, School of Biology, and Institute of Nutrition and Safety, University of Barcelona, Spain; 2 Enteric Virus Laboratory, Department of Cell Biology, Physiology and Immunology, School of Biology, University of Barcelona, Spain; 3 Liver Unit, Internal Medicine, Hepatic Diseases Laboratory, Vall d’Hebron Research Institute-Hospital Universitari Vall d’Hebron (VHIR-HUVH), Barcelona, Spain; 4 Centre of the Biomedical Research Network (CIBER) for Hepatic and Digestive Diseases (CIBERehd), Instituto de Salud Carlos III; 5 Roche Diagnostics SL, Barcelona, Spain

**Keywords:** codon usage, translation efficiency, sequence space, fitness landscape, genotype–phenotype maps

## Abstract

Hepatoviruses show an intriguing deviated codon usage, suggesting an evolutionary signature. Abundant and rare codons in the cellular genome are scarce in the human hepatitis A virus (HAV) genome, while intermediately abundant host codons are abundant in the virus. Genotype–phenotype maps, or fitness landscapes, are a means of representing a genotype position in sequence space and uncovering how genotype relates to phenotype and fitness. Using genotype–phenotype maps of the translation efficiency, we have shown the critical role of the HAV capsid codon composition in regulating translation and determining its robustness. Adaptation to an environmental perturbation such as the artificial induction of cellular shutoff—not naturally occurring in HAV infection—involved movements in the sequence space and dramatic changes of the translation efficiency. Capsid rare codons, including abundant and rare codons of the cellular genome, slowed down the translation efficiency in conditions of no cellular shutoff. In contrast, rare capsid codons that are abundant in the cellular genome were efficiently translated in conditions of shutoff. Capsid regions very rich in slowly translated codons adapt to shutoff through sequence space movements from positions with highly robust translation to others with diminished translation robustness. These movements paralleled decreases of the capsid physical and biological robustness, and resulted in the diversification of capsid phenotypes. The deviated codon usage of extant hepatoviruses compared with that of their hosts may suggest the occurrence of a virus ancestor with an optimized codon usage with respect to an unknown ancient host.

## Introduction

The human hepatitis A virus (HAV) is exceptionally stable to extreme conditions, including very low pH, high temperature, and desiccation, compared with other picornaviruses such as poliovirus and rhinoviruses (Reagan et al. 1981; Abad et al. 1994; Costafreda et al. 2014; Wang et al. 2015). In picornavirus capsids, the interface between the pentameric units determines the particle stability (Filman et al. 1989; Warwicker 1992). The recently resolved 3D structure of the HAV capsid revealed a VP2 domain swap at the pentamer interface creating a tighter subunit connectivity absent in other picornaviruses, and suggesting a relationship with its remarkable stability (Wang et al. 2015). However, this swap also occurs in the cricket paralysis virus (CrPV), a picorna-like insect virus, which does not show an equivalent stability (Wang et al. 2015). Consequently, the underlying mechanism of HAV capsid stability remains undefined.

HAV shows a unique codon composition avoiding the use of the most abundant host cell codons. Instead, abundant and rare host codons are rarely used in its genome, while intermediately abundant host codons are abundant in the viral genome (Pintó et al. 2018). Remarkably, this distinctive codon usage is very well conserved among the recently described hepatoviruses isolated from seals, marsupials, and small mammals (Anthony 2015; Drexler et al. 2015; de Oliveira Carneiro et al. 2018), in contrast with the markedly different pattern shown by other picornaviruses, suggesting an evolutionary signature. The driving force of this intriguing deviated codon usage may be the selection for a fine-tuned translation elongation (Aragonès et al. 2010). Rare codons in the capsid-coding region may slow down the translation elongation rate due to the translational sensitivity to tRNA availability (Wohlgemuth et al. 2013), and in doing so intrinsically modulate capsid folding. Unlike other picornaviruses, HAV is unable to induce cellular protein synthesis shutoff (Ali et al. 2001; Chase and Semler 2012). Interestingly, adaptation to artificially induced cellular shutoff involved dynamic codon changes which resulted in altered capsids (Costafreda et al. 2014). Similarly, adaptation of poliovirus to conditions of chaperon activity inhibition involved codon usage deviations at interdomain boundaries, likely promoting codon-derived cotranslational folding, and hence balancing the reduction of chaperon activity (Geller et al. 2018), altogether suggesting a role of codon composition in capsid folding and stability.

Robustness is defined as the invariance of phenotypes against perturbation (de Visser et al. 2003), and translational robustness has been defined as the protein folding robustness despite nonsynonymous mutations (Wilke and Drummond 2006; Drummond and Wilke 2008). Additionally, codon composition may also influence translation robustness and in turn folding robustness, which is critical for capsid stability of viruses transmitted through the fecal–oral route such as HAV.

Robustness may be mutational and environmental or in simple terms when different genotypes show the same phenotype we talk about mutational robustness, and when a genotype shows the same phenotype independently of the environmental conditions we refer to environmental robustness. However, the relationship between genotype and phenotype is very complex. This puzzle is starting to be solved by building fitness landscapes including genotype–phenotype (G → P) maps (Weinreich et al. 2006; Poelwijk et al. 2007; Draghi et al. 2010; Pigliucci 2010; Nichols et al. 2011; de Visser and Krug 2014). Fitness landscapes are highly appropriate for the study of the mutant networks defining virus populations (Hinkley et al. 2011; Wu et al. 2011; Lauring et al. 2012). In fact, RNA viruses due to their population size, their high mutation rate, their clonally reproducing system and their short generation time are ideal models for the study of robustness and adaptability (Burch and Chao 2000; Lauring et al. 2012, 2013).

The actual link between changes in codon usage (genotype) and translation efficiency (phenotype) is very difficult to establish, particularly at the population level. In the present study, we have used deep-sequencing to identify the HAV genotypes present in two populations long adapted to moderate and high levels of artificially induced cellular shutoff, respectively, compared with their parental population, and we have tested the translation pattern of each of these genotypes using a bicistronic system (Mueller et al. 2006). Through this approach, we have been able to build G → P maps for two genome fragments of the capsid differing in their codon composition and we have determined the translational robustness in different environments of cellular shutoff and its relationship with capsid folding robustness.

Finally, given the critical relationship between the codon usage and the robustness of the translational efficiency and capsid folding, we hypothesize the potential occurrence of an ancestor of the extant hepatoviruses with an optimized codon usage with respect to an unknown ancient host.

## Materials and Methods

### Relative Codon Deoptimization Index, tRNA Adaptation Index, and Theoretic Translation Elongation Rate

The Relative Codon Deoptimization Index (RCDI) estimates the match between the codon usage of a virus versus its host (Mueller et al. 2006). It is estimated following the formula RCDI  =  Σ [(C_*i*_×*F_a_*×*N_i_*/*C_i_*×*F_h_*)/*N*], where *C_i_F_a_* is the observed relative frequency of each codon (i) out of all synonymous codons for the same amino acid in the query sequence. *C_i_*F_*h*_ is the normal relative frequency observed in the host genome of each codon (i) out of all synonymous codons for that amino acid. *N_i_* is the number of occurrences of codon (i) in the sequence, and *N* is the total number of codons in the sequence. An RCDI = 1 indicates that a gene follows the host codon usage, while RCDI values >1 indicate deviations from the host codon usage. Additionally, the expected RCDI (eRCDI) was used as a threshold value to estimate whether the deviation is the product of compositional biases, with values over the eRCDI indicating selection (Puigbó et al. 2010). The eRCDI is computationally determined by generating random sequences with similar G + C and amino acid composition to the input sequences. The RCDI of these random-generated sequences enables to estimate the eRCDI using an upper one-sided tolerance interval for a normal distribution, which with a 95% confidence level will contain the 95% percentage of the sequence population. The upper limit represents the value not exceeded by the specified fraction of the randomly generated sequences (95%) with the chosen confidence limit (95%). The RCDI and eRCDI of the HAV, poliovirus (PV 1), human rhinovirus 14 (HRV 14), and human rhinovirus 89 (HRV 89) were calculated using the public server http://genomes.urv.es/CAIcal/RCDI/, last accessed July 22, 2019, following the standard human codon usage as the host reference. Moreover, the RCDI and eRCDI of the HAV capsid versus two subsets of genes differing in the level of expression in the liver was also figured. The mRNA copy numbers and the coding sequences of these genes were obtained from the Human Protein Atlas program (https://www.proteinatlas.org; last accessed July 22, 2019) and the Ensembl vertebrate genome browser (https://www.ensembl.org; last accessed July 22, 2019). The first subset of genes (*HP*, *SERPINA1*, *FGG*, *GC*, *ADH4*, *AHSG*, *HPX*, *VTN*, *SERPINC1*, and *CYP2E1*) with an average of 465 codons in length, was estimated to express an average of 7239 mRNA copy numbers per cell. The second subset (*CYP2A6*, *CPB2*, *C9*, *UGT2B4*, *AGXT*, *A1BG*, *C8B*, *F9*, *SLC38A4*, and *BAAT*) with a length of 486 codons was figured to express an average of 559 mRNA copies.

The tRNA adaptation Index (tAI) of a gene estimates the amount of adaptation to the tRNA pools and has been commonly used to measure the translation efficiency taking into account the intracellular concentration of tRNA molecules and the efficiencies of each codon–anticodon pairing (dos Reis et al. 2004; Dana and Tuller 2014). The tAI of the capsid of PV 1, HRV 14, HRV 89, and HAV were obtained using the stAIcalc calculator (Sabi et al. 2017) publicly available at http://tau-tai.azurewebsites.net/, last accessed July 22, 2019 using the human tRNA gene copy numbers obtained from http://gtrnadb.ucsc.edu/Hsapi19/Hsapi19-summary.html, last accessed July 22, 2019.

The theoretic translation elongation rate (*R*_c_) was assessed using a previously proposed algorithm (Shah and Gilchrist 2010). The rate of elongation of a codon (i) is estimated using the formula *R*_c_(i) = *a*×(Σ *t_j_*×*p*_c_×*w_j, i_*+ Σ *t_j_*×*p*_p_×*w_j, i_*), taking into consideration all their cognate (Σ *t*_j_×*p*_c_×*w_j, i_*) and pseudocognate (Σ *t_j_*×*p*_p_×*w_j, i_*) tRNAs. The parameter *t_j_* represents the gene copy number of *j*th tRNA species, and *w_j, i_* is the reduction in elongation probability due to wobble mismatch with values of 1.00 for A:U and G:C, 0.64 for U:G, G:U, A:C, and 0.60 for U:U, A:A, C:U, G:A, U:C, and A:G (Shah and Gilchrist 2010). Human tRNA gene copy numbers were obtained from http://gtrnadb.ucsc.edu/Hsapi19/Hsapi19-summary.html, last accessed July 22, 2019 and Iben and Maraia (2014). The parameters *a*, *p*_c_, and *p*_p_, represent the scaling constant between tRNA gene copy number and elongation rate, the probability of elongation by cognate tRNA per tRNA entry, and the probability of elongation by pseudocognate tRNA per tRNA entry, respectively. The *p*_c_ = 0.652 and *p*_p_ = 0.00062 parameters were obtained from (Shah and Gilchrist 2010). The *a* parameter was calculated as the value necessary to adjust the average of the theoretical rate of elongation of all the codons in *Homo sapiens* (9.93 aa/sec) to the actual values obtained in experimental work. Data of the actual rate of elongation in eukaryotic cells and particularly from different human tissues are scarce. In a recent work on organ-specific translation elongation rates measured in vivo (Gerashchenko et al. 2018), elongation rates of 6.8 aa/sec and 4.4 aa/sec for the liver and the skeletal muscle, respectively, are described. The liver is the target tissue for HAV, giving an *a* = 0.6855. We found no data for intestinal cells or respiratory cells, the major targets for PV and HRV, respectively. However, since the speed of translation has been related to the metabolic activity of the tissues, and this activity is reported to be similar between the intestinal cells, the respiratory cells and the skeletal muscle (Wang et al. 2012), we adopted the elongation rate reported for the skeletal muscle to estimate an *a* = 0.4435 for PV and HRV. The *R*_c_ of genome fragments was estimated as the average of the *R*_c_ of their codons.

The RCDI, tAI, and *R*_c_ algorithms, altogether, provide a more reliable indication of the rate of translation than each of them separately, and were analyzed along the capsid-coding region by using sequence windows of 100 codons width. These windows were moved over the sequence in steps of 15 codons. The RCDI, tAI, and *R*_c_ of each of these windows was calculated, and the mean and SD was estimated. The one-way ANOVA was used to look for significant differences between these means from the different viruses using the SigmaPlot v11, which includes normality and equal variance tests and when they fail moves to the Kruskal–Wallis on Ranks; the Tukey’s test is used for pair-wise comparisons.

### Cells and Viruses

Three previously characterized HAV populations were used throughout this study, including population L0, adapted to conditions of no cellular shutoff, population F0.05LA, adapted to conditions of moderate cellular shutoff, and population F0.2LA adapted to conditions of high cellular shutoff (Costafreda et al. 2014).

Viruses were grown in fetal rhesus monkey kidney-4 (FRhK-4) cells. For populations F0.05LA and F0.2LA, actinomycin D (AMD, Sigma), at concentrations of 0.05 and 0.20 µg/ml was added in the postinfection media to inhibit cell DNA transcription at moderate levels (∼60–70%), and at high levels (>90%), respectively (Aragonès et al. 2010). L0 population was mutagenized using 5-fluorouracyl (FU). FRhK-4 cells were preincubated with 80 µM FU for 18 h prior to infection, followed by the infection in the presence of the same drug concentration. All attempts to mutagenize populations F0.05LA and F0.2LA were unsuccessful due to the additive toxic effects of AMD and FU combined with the long times required for HAV replication.

### Ultra-Deep PyroSequencing and Analysis of the Mutant Spectra

Ultra-deep pyrosequencing (UDPS) (454 GS-Junior Life Sciences, Roche) of two fragments of the VP3 (nt 1474–1838) and VP1 (nt 2394–2852) coding regions, including sequences from both DNA strands, were analyzed using a multiplex format based on the use of primers including a universal M13 sequence and a multiplex identifier (MID) sequence (Quer et al. 2015). Reverse (with the M13 universal sequence underlined) and forward primers used were as follows: 5′CACAGGAAAC AGCTA TGACCGGGAAAA ACTTGA A A ATC AAAGAC3′ and 5′GTTGTAAAA CGACGGCCAG TGAG A AAT GAATTTAGGGTCAG3′ for VP3 and 5′CACAGG AAACAGCTATGACCCAGTGCTCCAGA CACAGC3′ and 5′G T TG TAAAACGAC GGCCAGTAAA GTRCCTGAGACA TT TC C T G3′ for VP1.

HAV RNA was extracted from 150 µl cell culture supernatants with NucleoSpin RNA Virus kit (Macherey-Nagel), according to the manufacturer’s instructions, and eluted in 50 µl. About 5 µl of RNA were used in the RT-PCRs, which were performed using the Expand Reverse Transcriptase (Roche) and the high-fidelity *Pwo* DNA polymerase (Roche). The High Pure PCR Product Purification Kit (Roche) was used for the purification of PCR products. A reamplification for the addition of the MID signaling was required and the DNA was purified again. The quality and quantity of the DNA was tested using the BioAnalyzer DNA 1000 LabChip (Agilent) and the PicoGreen assay (Invitrogen) and sequences were obtained using the GS-Junior Titanium Sequencing kit.

Analysis of sequences were made using a previously described pipeline (Gregori et al. 2013, 2014; Quer et al. 2015). Briefly, the total sequence-containing fasta file was demultiplexed to obtain a fasta file for each sample and strand. Reads not identified by MID and/or primer were discarded. More than two mismatches in the specific sequence, three on the universal M13 sequence and one on the MID sequence or the presence of an indel in the reads were not accepted. Sequences showing >2 *N*s or 3 gaps or not covering the full amplicon were also discarded. Sequences not observed on the forward and reverse strands were discarded. Genotype frequencies were computed as the number of observed reads excluding those with frequencies <0.5%.

The mutant spectra characterization included the rate of synonymous mutations per synonymous site (*K*_s_), the rate of nonsynonymous mutations per nonsynonymous site (*K*_a_) and the normalized Shannon entropy (*S*_n_) as a measure of the genotype diversity. *K*_s_ and *K*_a_ were calculated with the DnaSP program (http://www.ub.es/dnasp/; last accessed July 22, 2019). For a given population, each genotype was compared with all other genotypes, obtaining a *K*_s_ and *K*_a_ mean for each genotype. These means were normalized by the genotype frequency to figure out the average of the *K*_a_, *K*_s_, and *K*_a_/*K*_s_ of each fragment in each population (Sabrià et al. 2019). The 95% confidence interval, using the Bonferroni method for samples with thousands of repetitive values, was used to look for significant differences of each parameter between populations and fragments and between parameters in a given fragment and population (https://onlinecourses.science.psu.edu/stat100; last accessed July 22, 2019). *S*_n_ was calculated following the formula $S_{n}=-\left[\sum_{i=1}^{h}(p_{i}\times \ln p_{i})\right]/\ln N$, where *p_i_* is the frequency of each genotype, *h* the number of observed genotypes, and *N* is the total number of sequences in the spectrum of mutants (Airaksinen et al. 2003). *S*_n_ ranges from 0 (no diversity) to 1 (maximum diversity).

The proportion of mutations in the swarm of mutants increasing or decreasing the *R*_c_ in each fragment was calculated and compared with the expected values. These expected values were figured from all possible sense mutations that could be generated taking into consideration the codon composition of each fragment. The differences between the observed and expected values were evaluated using the χ^2^ test for the goodness of fit through a contingency 2×2 table to compute *P* values (https://www.socscistatistics.com/tests/goodnessoffit/default2.aspx, last accessed July 22, 2019).

### In Vitro Assays for the Determination of the Translation Efficiency

Cloning of the different genotypes of VP3 and VP1 fragments in the bicistronic G1RCMsKp and G1RCMsKp-mut vectors (Pérez-Rodríguez et al. 2016) was performed. The G1RCMsKp-mut vector harbors three mutations (U359C, U590C, U726C) which enhance its activity in directing translation. These vectors show cap-dependent translation of the *Renilla reniformis* luciferase gene (RLuc) and HAV IRES-dependent translation of the *Photinus pyralis* (firefly) luciferase gene (FLuc). Both fragments were amplified from viral populations by RT-PCR using the following primers:

VP3KpnI-5′CCCGGTACC GGGAAAAACTTGAA AAT CA A A GAC3′ and 5′VP3CCCCATGATGAGAAATGAATTT AGG GT CAG-3′ for VP3 and VP1KpnI-5′CCCGGTACCT GAT TGTTC TGTGACA GA C A AATAACAACT3′ and VP1CC5′P  + 5′C CTTT CCTGAA TTGAAACCTGGAGAATCC3′ for VP1. Viral RNA extraction, RT-PCRs, and DNA purification were performed as above described but using 10 µl of a 1/10 diluted RNA in the RT reaction. For cloning, the vector was digested with the *Kpn*I and *Msc*I restriction enzymes and treated with the FastAP thermosensitive alkaline phosphatase (Thermo Scientific). Amplified fragments were digested with the *Kpn*I enzyme. Vector and fragments were overnight ligated using the T4 DNA ligase (Thermo Scientific) and transformed into MegaX DH10B T1 Electrocomp Cells (Invitrogen).

The translation efficiency of the molecular clones corresponding to the different genotypes (supplementary tables S1 and S2, Supplementary Material online) was tested. Monolayers of FRhK-4 cells grown in 96-well microtiters were transfected with the different vectors as previously described (Pérez-Rodríguez et al. 2016). Vector DNA was resuspended in Opti-MEM I (Thermo Fisher Scientific) to a concentration of 0.01 µg/µl and X-tremeGENE HP DNA and Transfection Reagent (Roche) added at a 4% (v:v) concentration. After 15-min incubation, 25 µl of this suspension was added per well. Cells were incubated for 30 min and finally 60 µl of posttransfection medium (Opti-MEM I) added. The bioluminescence activity was measured with the Dual-Glo Luciferase Assay System (Promega), and detected with a luminometer (Lumat LB 9507, Berthold Technologies). Light emission was measured 10 min after addition of each substrate and integrated over a 10-s interval. Having in mind the low efficiency of the HAV IRES, a first experiment including two replicas to find the optimal time for bioluminescence reading (18, 21, 24, and 30 h) was performed. Readings at 24 h proved to be a robust indication of the translation efficiency, and were used thereafter along the study (supplementary fig. S1, Supplementary Material online). Three different experiments, including two replicas each, for each genotype-derived vector and for each growing condition were performed. Additionally, as negative controls, cells transfected with the digested vector alone were included.

The ratio FLuc/RLuc at 24 h of incubation after transfection was used as an indication of the efficiency of translation normalized versus the transcription shutoff and the efficiency of transfection. The one-way ANOVA–Tukey’s test was used to distinguish between the ratios shown by each genotype using the R package. This test allows classifying the treatments (translation efficiency of each genotype) in a gradient of letters. Statistically significant differences are depicted by different letters or combinations of letters (a=ab, b=ab but a ≠ b and so on).

Relative translation efficiency was estimated as the average and standard error of the fold increase of the FLuc/RLuc ratio of all measures from each genotype and condition, relative to the average of the FLuc/RLuc ratio of the most abundant genotype in the L0 population in the absence of shutoff. These normalized data were used to distinguish between different phenotypes by the one-way ANOVA–Tukey’s test. Distinct phenotypes were defined as those showing different letters or different combination of letters. For instance, although a=ab and b=ab, a ≠ b, implying the occurrence of three phenotypes.

### RNA Secondary Structure Estimation

The pairing number (P-num) provides a quantitative estimation of the propensity of a base to pair with alternative partners in a collection of suboptimal folds (Jaeger et al. 1989; Palmenberg and Sgro 1997; Zuker and Jacobson 1998). The lower the P-num value the higher the secondary structure. P-num values of the RNA from the different genotypes were figured using the public server http://mfold.RNA.albany.edu/? q=mfold; last accessed July 22, 2019.

### Genotype–Phenotype (G → P) Maps, Translation Robustness, and Phenotype Accessibility

To estimate the robustness of the translation efficiency and phenotype accessibility of the populations under study in the different shutoff conditions, we built G → P maps as previously described (Draghi et al. 2010). The phenotypic neighborhood of a genotype is constituted by its accessible one mutation away phenotypes. Genotype robustness (*q*) is estimated considering the proportion of neighbor genotypes (one mutation away) showing the same phenotype. The phenotype accessibility (*K*/*P*) of a genotype is estimated as the proportion of accessible phenotypes (one mutation away; *K*) among all phenotypes (*P*) present in the whole landscape. Robustness (*q*) and phenotype accessibility (*K*/*P*) at the population level are defined as the average (taking into consideration the frequency of the genotypes obtained through deep-sequencing) of the robustness and accessibilities of each individual genotype.

Again, the 95% confidence interval using the Bonferroni method was used to look for significant differences of *q* and *K*/*P* between conditions, populations, and fragments.

### Virus Behavior under Different Physiological and Physical Conditions

Resistance to 300 MPa high hydrostatic pressure (HHP) for 1 min at 10 °C, and to pH 2 for 1 h at 37 °C was tested as a measure of the capsid fragility and acid denaturability, respectively. To quantify virus decay, nontreated controls were run in parallel and used for the calculation of the log_10_ reduction of virus titers (LTR). The 50% uncoating time (UT_50_) was determined as a measure of the capsid flexibility, using the classical method of virus replication in the presence of neutral red (Costafreda et al. 2014). A regression line between the percent titer reductions caused by neutral red compared with a control kept always in the dark, and the time in minutes when samples were lit up was drawn. The UT_50_ was inferred from this regression line. Four different experiments were performed for each parameter, with two titrations of all samples. To avoid interassay variabilities, LTRs and UT_50s_ were calculated in 4 (nontreated) by 4 (treated) combinations. Infectious virus titers (TCID_50_) were obtained in FRhK-4 cell monolayers in the absence of AMD. Box-and-whisker plots were used to analyze the diversity of behaviors. For each condition and population, the distance between the higher and lower values was used as an indication of the length of the box-whisker plot. Statistical differences between these values in the different populations were determined using the χ^2^ statistic through a contingency 2×2 table to compute *P* values (https://www.socscistatistics.com/tests/chisquare/default2.aspx; last accessed July 22, 2019).

### Codon Usage Matrix

A codon usage-based Euclidean distance matrix (Nesti et al. 1995) between several picorna-like viruses and a range of eukaryotic organisms from protozoa to animals including platyhelminthes, annelids, nematodes, molluscs, insects, aves, and mammals was built using the DendroUPGMA program (http://genomes.urv.cat/UPGMA/; last accessed July 22, 2019). For more information on viruses and host included in the analysis, see supplementary figure S6, Supplementary Material online, legend. The codon usage of all these organisms was available at the Codon Usage Database (http://www.kazusa.or.jp/codon/; last accessed July 22, 2019), and was based in a significant number of sequenced genes. The obtained matrix was used to build a heat map using the Python library Matplotlib.

## Results

### Codon-Derived Parameters in HAV

The RCDI of the capsid coding region of the HAV, compared with several picornaviruses including PV 1, HRV 14 and HRV 89 was estimated with respect the standard human codon usage (table 1). Only in HAV the RCDI was above the eRCDI threshold, indicating that its codon usage deviation is not only due to compositional biases. Additionally, for HAV the RCDI with regard the codon usage of two subsets of genes differing in their expression level in the liver was also calculated. Similar levels of deviation were observed (table 1). Hence, the standard human codon usage was used as the reference in the subsequent analyses.

**Table 1:** Relative Codon Deoptimization Index of Poliovirus 1 (PV 1), Hepatitis A Virus (HAV), Human Rhinoviruses 14 (HRV 14), and 89 (HRV 89), with Respect the Standard Human Codon Usage and of Different Subsets of Genes

Codon Usage	PV 1		HRV 14		HRV 89		HAV	
	RCDI^a^	eRCDI^a^	RCDI^a^	eRCDI^a^	RCDI^a^	eRCDI^a^	RCDI^a^	eRCDI^a^
Standard	1.133	1.418	1.133	1.414	1.424	1.461	1.687	1.517
Highly expressed genes in liver	NA^b^	NA^b^	NA^b^	NA^b^	NA^b^	NA^b^	1.665	1.598
Lowly expressed genes in liver	NA^b^	NA^b^	NA^b^	NA^b^	NA^b^	NA^b^	1.583	1.510

a When RCDI > eRCDI indicates that the deviation is not due to compositional biases.

b NA, since only HAV infects the liver.

RCDI, Relative Codon Deoptimization Index; eRCDI, Expected RCDI; NA, not applicable.

**Fig. 1.**
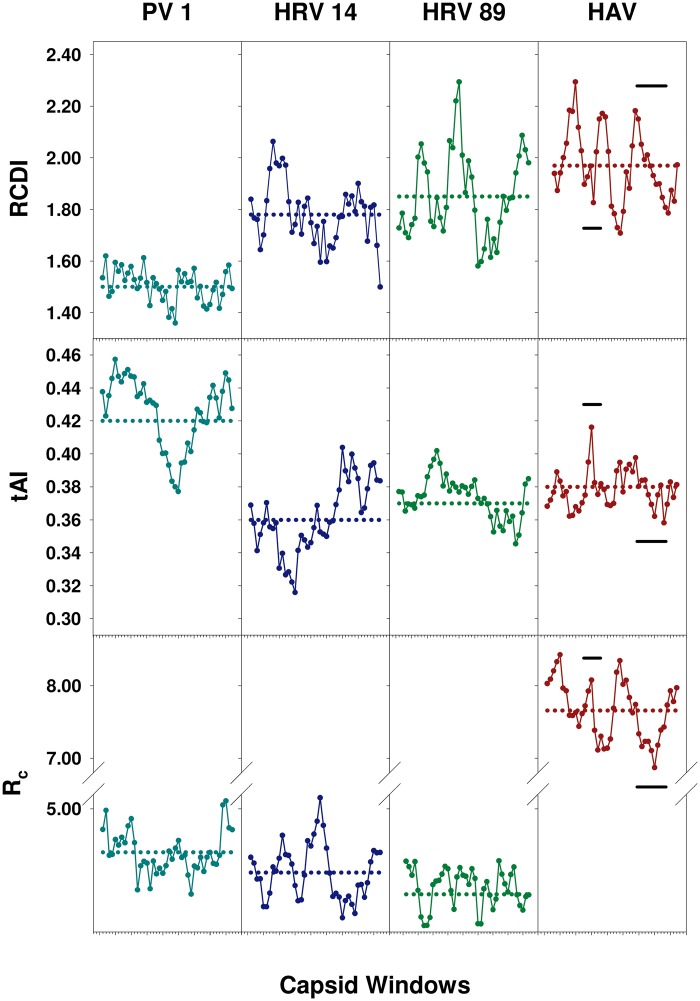
—Relative Codon Deoptimization Index (RCDI), tRNA Adaptation Index (tAI), and translation elongation Rate (*R*_c_) of the capsid of several picornaviruses. PV 1 (poliovirus 1) is depicted in turquoise, HRV 14 (human rhinovirus 14) in violet, HRV 89 (human rhinovirus 89) in green, and HAV (human hepatitis A virus) in brown. Analyses were performed in 100 codon length windows sliding every 15 codons. Doted horizontal lines depict the average from all the windows. Regarding RCDI and tAI, PV 1 significantly differed (*P* < 0.001) from the rest of viruses and HRV 14 also differed significantly (*P* < 0.001) from HAV. *R*_c_ was significantly higher (*P* < 0.001) in HAV, and in PV 1 was also significantly higher (*P* < 0.001) than in HRV 14 and HRV 89. The HAV fragments under study are indicated with black solid lines.

The RCDI, the tAI, and the theoretical translation elongation rate (*R*_c_) along the capsid coding region of HAV, PV 1, HRV 14, and HRV 89 was estimated. These analyses were performed using overlapping fragments to identify capsid regions with high levels of codon deviation, and low levels of codon adaptation and elongation rates. The average of the RCDI of all the overlapping fragments progressively and significantly (*P* < 0.001) increased from PV 1, to HRV 14 and HRV 89 and consequently the tAI and the *R*_c_ average significantly (*P* < 0.001) decreased (fig. 1). However, HAV behaved as an exception showing the highest RCDI (*P* < 0.001), and unexpectedly the highest *R*_c_ (*P* < 0.001) as well (fig. 1). The unexpected relationship in HAV could be related to the fact that the *R*_c_ algorithm does not take into account the competition for the tRNAs between the virus and the cell. This scenario may be particularly relevant for HAV, which is unable to induce the cellular protein synthesis shutoff compared with PV and HRVs that do induce it (Ali et al. 2001; Walsh et al. 2013).

### Environmental Perturbations Shape Translation Landscapes and Sequence Space

The RCDI, tAI, and *R*_c_ algorithms attempt to predict the relationship between codon usage and the translation efficiency, when data on the actual tRNA pools are not available. Being this a very complex prediction, in virus-infected cells it is even worse due to the associated and unknown changes in the tRNA pools. In consequence, we attempted to understand the underlying reasons of the deviated codon usage of HAV by performing experimental evolution under changing conditions of host tRNA demand, and hence altering the tRNA pools available for the virus.

Having in mind the critical role that tRNA availability plays on the translation efficiency (Zhang et al. 2009; Fedyunin et al. 2012; Wohlgemuth et al. 2013; Weinberg et al. 2016), and the codon frequency-driven adaptability of HAV to conditions of artificially induced transcription shutoff (Aragonès et al. 2010; Costafreda et al. 2014), we decided to undertake an analysis of the translation efficiency of the HAV mutant swarm.

Two capsid coding fragments located in a *R*_c_ peak and in a *R*_c_ valley (fig. 1), and more important, showing different codon composition were used for the study (table 2): a fragment from the VP3 coding region representing 50% of the total VP3 length, and a fragment from the VP1 coding region representing 46% of the total VP1 length.

**Table 2:** Characteristics of the Capsid Fragments under Study

	VP3	VP1
Codon positions (length)	1–123 (123)	71–209 (139)
RCDI/eRCDI^a^	1.94/1.52	1.72/1.49
*R*_c_	11.60	10.60
Frequency of the most common codon for each amino acid (*Homo sapiens*) (%)	19	28
Frequency of the least common codon for each amino acid (*Homo sapiens*) (%)	15	23

a When RCDI > eRCDI indicates that the deoptimization is not due to compositional biases.

RCDI: Relative Codon Deoptimization Index; eRCDI, Expected RCDI; *R*_c_: translation elongation rate.

**Fig. 2.**
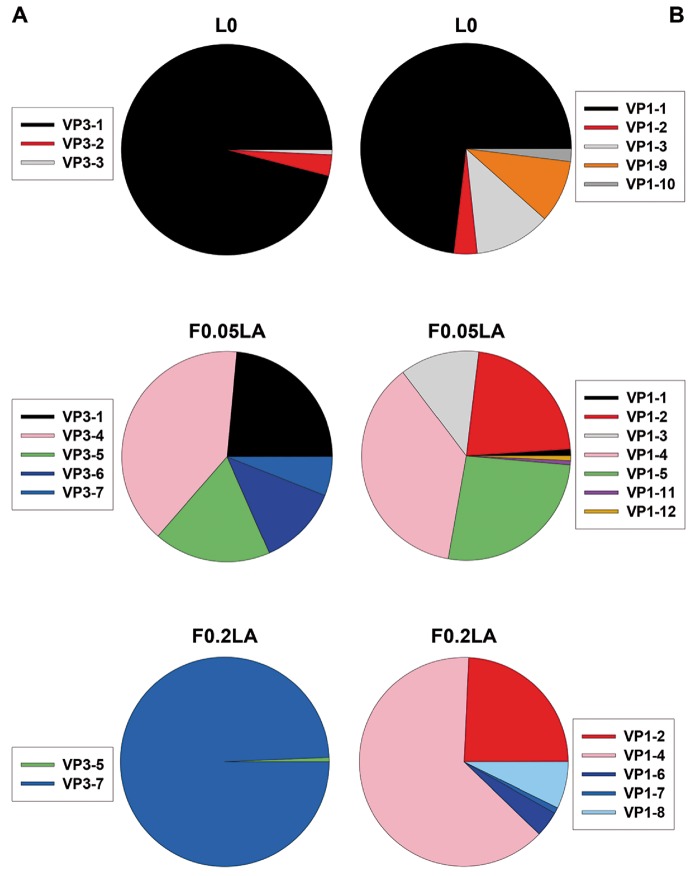
—Analyses of the mutant spectra. Genotype distribution of VP3 (*A*) and VP1 (*B*) in populations L0, F0.05LA, and F0.2 LA.

First, a deep-sequencing analysis was performed to assess the diversity of three HAV populations, which differed in their capacity to grow in conditions of shutoff. The L0 parental-type population was adapted to grow in conditions of no shutoff for five passages. The F0.05LA population was adapted to grow in conditions of moderate shutoff induced by 0.05 µg/ml of AMD for 120 passages, and the F0.2LA population was adapted to grow in conditions of high shutoff induced by 0.20 µg/ml of AMD for 70 passages, additionally to a preadaptation to moderate shutoff for 60 passages. This analysis revealed the occurrence of seven different genotypes among the mutant spectra of the VP3 fragment (supplementary table S1, Supplementary Material online) with 3 (from a total *n* = 7,826 sequences), 5 (*n* = 6,835), and 2 (*n* = 7,418) of them occurring in populations L0, F0.05LA, and F0.2LA, respectively (fig. 2A), resulting in entropy values (*S*_n_) of 0.02, 0.16, and 0.00 (table 3). In contrast, 12 different genotypes were detected in the VP1 fragment (supplementary table S2, Supplementary Material online), with 5 (*n* = 3,370), 7 (*n* = 3,120), and 5 (*n* = 2,860) of them in populations L0, F0.05LA, and F0.2LA (fig. 2B), with a subsequent *S*_n_ of 0.11, 0.18, and 0.12, respectively (table 3). The mutant swarms of L0 population after 103 passages in the absence of AMD were similar to the passage five swarms, and different from the F0.05LA or F0.2LA populations (supplementary fig. S2, Supplementary Material online and fig. 2).

**Table 3:** Analysis of the Mutant Spectra

	VP3				VP1			
	*K*_s_ ^a^	*K*_a_^a^	*K*_a_^a^/*K*_s_^a^	*S*_n_	*K*_s_ ^a^	*K*_a_^a^	*K*_a_^a^/*K*_s_^a^	*S*_n_
L0	0.0099 a, A, α	0.0055 a, B, α	0.559 a, α	0.02	0.0048 a, A, β	0.0061 a, B, β	1.293 a, β	0.11
F0.05LA	0.0144 b, A, α	0.0086 b, B, α	0.713 b, α	0.16	0.0047 a, A, β	0.0051 b, B, β	1.097 b, β	0.18
F0.2LA	0.0098 a, A, α	0.0067 c, B, α	0.686 c, α	0.00	0.0055 b, A, β	0.0053 b, B, β	1.067 c, β	0.12

Note.—Genetic features include the rate of synonymous mutations per synonymous site (*K*_s_), the rate of nonsynonymous mutations per nonsynonymous site (*K*_a_), and the normalized Shannon entropy (*S*_n_).

a Statistically significant differences (*P* < 0.05) are depicted following the criteria of different letters: lowercase letters refer to differences of *K*_s_, *K*_a_, or *K*_a_/*K*_s_ between populations in a fragment, uppercase letters refer to differences between *K*_s_ and *K*_a_ in a population and fragment, Greek letters refer to differences between *K*_s_, *K*_a_, or *K*_a_/*K*_s_ in a population between fragments.

Remarkably, the rate of synonymous mutations per synonymous site (*K*_s_) and the rate of nonsynonymous mutations per nonsynonymous site (*K*_a_) differed significantly (*P* < 0.05) between populations and fragments (table 3). Additionally, in L0 population and in VP1, *K*_a_ was significantly higher (*P* < 0.05) than *K*_s_. This unexpected result could be related to the need to maintain the codon frequencies, which is facilitated through nonsynonymous mutations (supplementary table S3, Supplementary Material online). In fact, when L0 population was mutagenized with 80 µM 5-fluorouracil (FU), the same tendency was observed (supplementary table S4, Supplementary Material online). In contrast, in populations adapted to shutoff, *K*_a_ significantly decreased (*P* < 0.05) compared with L0, and consequently the *K*_a_/*K*_s_ ratio decreased as well (table 3). Similarly, this result may be related to the need, in conditions of shutoff, to change the codon frequencies, which is more likely to occur through synonymous mutations (supplementary table S3, Supplementary Material online). Unfortunately, attempts to mutagenize populations adapted to shutoff were unsuccessful. In VP3, *K*_s_ was significantly higher (*P* < 0.05) than *K*_a_ (table 3 and supplementary table S4, Supplementary Material online) suggesting a less restrictive need to maintain the codon frequencies in this region.

Second, to assess the potential influence of the mutations selected during the process of adaptation to conditions of cellular shutoff on the translation rate, the associated variations of the *R*_c_ were analyzed. Overall, the results proved that while in VP1 72% of the mutations reduced the *R*_c_, significantly over the expected values (*P* = 0.00001), in VP3 only 58% of mutations induced such a decrease (table 4 and supplementary tables S1, S2, and S5, Supplementary Material online). Remarkably, none of the mutations detected in the VP1 swarm of mutants of populations adapted to AMD, and almost none in the VP3 fragment, not even the dominant ones, have ever been observed in the consensus sequences of the field strains available in GenBank (AAA45465-45466, AAA45471, AAK44219, AAL91576, AAM08224, AAQ08056, AAU87586, ABX44727, AEN93981, AKI05745, BAF63620-63623, BAK82406, BAO04548-04549, BAO09699-09704, BAQ84114, M14707). Altogether, it would suggest the action of selection to modulate the speed of translation in the new environmental conditions.

**Table 4:** Effect of Mutations Detected during the Process of Adaptation to Conditions of Cellular Shutoff on the Theoretic Translation Elongation Rate (*R*_c_)

Mutations Increasing and Decreasing the *R*_c_ per Fragment			
VP3		VP1	
**Δ↑**	**Δ↓**	**Δ↑**	**Δ↓**
42 (37)	58 (55)	28 (46)	72 (46)

Note.—Figures represent the percent of total mutations, which increase Δ**↑** or decrease Δ↓ *R*_c_. The expected percent is shown in brackets, the difference up to 100% correspond to mutations with no influence on the *R*_c_.

**Fig. 3.**
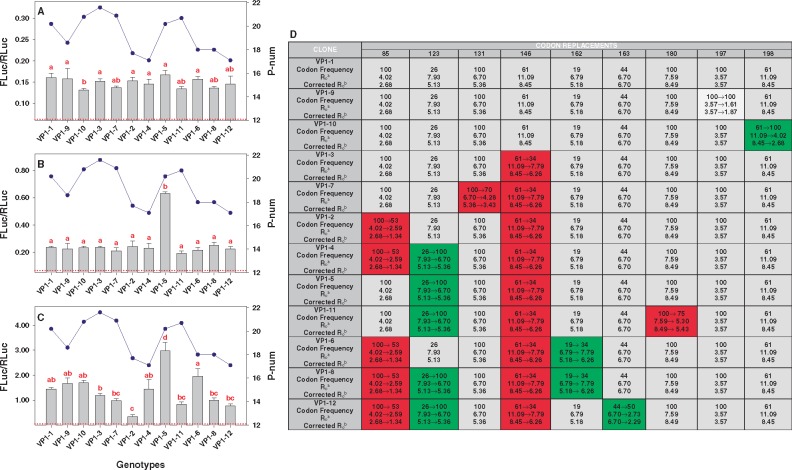
—Effect of mutations inducing changes in the codon frequencies in the translation rate. The actual translation efficiency of the VP1 clones is shown. Results are based on three different experiments, each including two replicas. (*A*) Conditions of no shutoff, (*B*) conditions of moderate shutoff, and (*C*) conditions of high shutoff. The dotted red line correspond to the average of a negative control corresponding to cells transfected with the digested vector alone. A new pdf file, corresponding to panels A, B and C, has been uploaded since the dotted red line in panel A is not visible. Statistically significant differences (*P* < 0.05) are depicted by different combinations of letters (ab=a, ab=b; bc=b, bc=c; a ≠ b, a ≠ c, b ≠ c; a ≠ d, b ≠ d, c ≠ d). The blue line plots represent the P-num, which predicts the potential occurrence of secondary structures in the RNA of the different genotypes; the lower the P-num value the higher the secondary structures in the RNA. (*D*) Information on the codon replacements present in each clone, compared with the most abundant genotype (VP1-1). Red and green background colors represent a change to a less and more frequent codon, respectively, and light gray a mutation not changing the codon frequency. ^a^Theoretical translation elongation rate (*R*_c_) of codons calculated using the data on human tRNA copy numbers available in http://gtrnadb.ucsc.edu/Hsapi19/Hsapi19-summary.html, last accessed July 22, 2019. ^b^*R*_c_ values corrected based on tRNA copy numbers available in (Iben and Maraia 2014).

To confirm the actual impact of codon composition on the regulation of the overall translation rate, we analyzed the translation efficiency in the swarm of mutants. These analyses revealed that codon composition had remarkable consequences, with genotypes differing in very few mutations showing dramatic changes in their efficiency of translation. As an example, the actual translation rates of the VP1 clones are shown in figure 3. Three different experiments, each including two replicas were performed. Translation efficiency was tested in conditions of no shutoff (fig. 3A), moderate shutoff (fig. 3B), and high shutoff (fig. 3C). Clones VP1-9, VP1-10, and VP1-3 differed by a single mutation with respect to the most abundant genotype, VP1-1, of the parental population. VP1-9 harbored a nonsynonymous mutation (supplementary table S2, Supplementary Material online), which reduced the *R*_c_ but did not change the codon frequency compared with VP1-1 (fig. 3D), and no significant differences in the actual rate of translation were observed (fig. 3A–*C*). In contrast, VP1-10, which harbored a synonymous mutation (supplementary table S2, Supplementary Material online) reducing the *R*_c_ but increasing the codon frequency (fig. 3D), showed a significant decrease in the actual rate of translation only in conditions of no shutoff (fig. 3A–*C*). Finally, VP1-3 harbored a nonsynonymous mutation (supplementary table S2, Supplementary Material online) inducing a reduction of the codon frequency and the *R*_c_ (fig. 3D), but no differences in the actual rate of translation were observed (fig. 3A–*C*).

Four additional clones differing by a single mutation compared with VP1-3 (VP1-7, VP1-2, and VP1-5) were also analyzed. VP1-7 harbored a synonymous mutation compared with VP1-3 (supplementary table S2, Supplementary Material online), that reduced both the codon frequency and the *R*_c_ (fig. 3D), but no reduction of the actual translation rate was observed (fig. 3A–*C*). VP1-2 harbored a nonsynonymous mutation, with respect to VP1-3 (supplementary table S2, Supplementary Material online), that induced a reduction of both the codon frequency and the *R*_c_ (fig. 3D). A significant reduction on the actual speed of translation in conditions of high shutoff was observed (fig. 3D). Finally, VP1-5 harbored a nonsynonymous mutation, compared with VP1-3 (supplementary table S2, Supplementary Material online), inducing an increase of the codon frequency and no change/reduction of the *R*_c_ depending on the tRNA database used (fig. 3D). An increase of the actual translation rate was observed in conditions of shutoff, not only compared with VP1-3 but to any other genotype (fig. 3A–*C*). Remarkably, VP1-4, that harbored this same mutation, showed a significantly lower translation rate in conditions of shutoff compared with VP1-5 (fig. 3A–*C*), thanks to an additional nonsynonymous mutation (supplementary table S2, Supplementary Material online) inducing a decrease in the codon frequency and the *R*_c_ (fig. 3D). Similarly, VP1-11 that harbored an additional synonymous mutation lowering the codon frequency and the *R*_c_, compared with VP1-5, also showed a decrease of the translation rate in conditions of shutoff. The VP1-2 clone harboring two mutations decreasing the codon frequency and the *R*_c_ compared with VP1-1, and lacking any compensatory mutation as it happened in other clones such as VP1-4, VP1-6, VP1-8, and VP1-12 (fig. 3D), was the genotype showing the lowest translation rate in conditions of shutoff (fig. 3A–*C*). The general lack of correlation between conditions of no shutoff and shutoff may be related to the fact that the most common codons are not as efficiently translated in the absence of shutoff, which may be related to the competition for the tRNAs (Pintó et al. 2018). Similar conclusions were obtained with the VP3 clones (supplementary fig. S3, Supplementary Material online).

Nevertheless, despite these amazing effects of codon composition on the firefly protein yield, other factors such as transcription and translation initiation and the mRNA stability (Bazzini et al. 2016; Rodnina 2016) may also have played a role. The bicistronic system used normalizes the global translation efficiency (FLuc) versus the global transcription efficiency (RLuc), including both their initiation and their rates. However, to prove whether the inefficiency of the HAV IRES may have influenced the FLuc/RLuc ratios, genotypes showing significant different behaviors under specific shutoff conditions (no shutoff and high shutoff for VP3 and VP1, respectively) were tested using a mutated and more active form of the HAV IRES (Pérez-Rodríguez et al. 2016). The same translation patterns were observed independently of the IRES-type (supplementary fig. S4, Supplementary Material online), although the global FLuc/RLuc ratios significantly increased (*P* < 0.03) with the more active IRES. These results indicate that translation initiation is not a main factor explaining the different genotype behaviors. Regarding the mRNA stability, it has been proposed that the occurrence of thermodynamically stable elements, such as pseudoknots and stem loops, slow down the ribosome movement along the mRNA (Caliskan et al. 2014; Belardinelli et al. 2016), sometimes in an environmental-depending manner (Frolov and Schlesinger 1994). We analyzed the presence of potential secondary structures in the RNA of the different genotypes using the P-num algorithm. RNA regions having abundant bases with low P-num values are predicted to contain secondary structures. No correlation was found between the lower P-num (highly structured) and a lower FLuc/RLuc ratio either in the VP1 genotypes (fig. 3) or in the VP3 genotypes (supplementary fig. S3, Supplementary Material online). Additionally, while the RNA structure is expected to be the same independently of the shutoff conditions, great differences on the FLuc/RLuc ratios were detected depending on these conditions. Altogether indicates that the mRNA structure is not a main factor underlying the different protein yields between genotypes. Finally, it has also been proposed that codon composition by itself may influence mRNA degradation (Bazzini et al. 2016). Interestingly, we cannot rule out in our genotypes the possibility that the rare codons could drive RNA degradation more efficiently than the optimal codons, and this degradation in combination with a slower translation rate would render a significantly lower global translation efficiency and protein yield.

The translation efficiency fold increase of each individual genotype compared with the average of the translation efficiency of the most abundant clone from population L0 (VP3-1 and VP1-1) in conditions of no shutoff was figured and the ANOVA–Tukey’s test used to define different phenotypes (supplementary tables S5 and S6, Supplementary Material online).

**Fig. 4.**
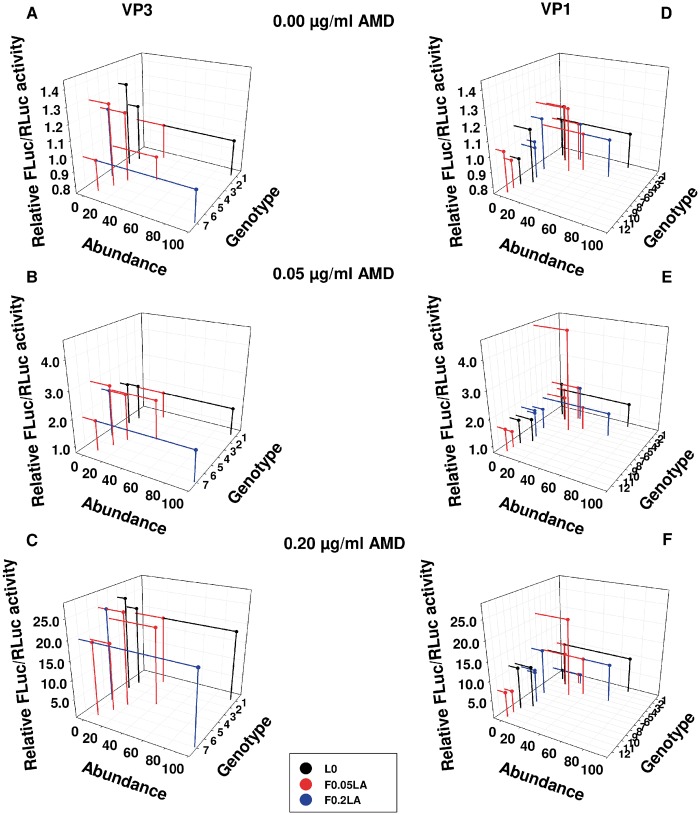
—Translation efficiency landscapes. (*A*), (*B*), and (*C*) panels show the VP3 landscape, and (*D*), (*E*), and (*F*) panels show the VP1 landscape. (*A*) and (*D*) panels include the landscape in absence of cellular shutoff (0.00 µg/ml of AMD), (*B*) and (*E*) panels include the landscape in conditions of moderate cellular shutoff (0.05 µg/ml of AMD, and [*C*] and [*F*] panels in conditions of high cellular shutoff [0.20 µg/ml AMD]). The landscapes were built by representing the relationship between three parameters: each genotype, their Relative FLuc/RLuc activity (phenotype) and its abundance (percentage). Black dots correspond to the L0 genotypes, red dots to the F0.05LA genotypes and blue dots to the F0.2LA genotypes.

Combining the information on the translation efficiencies, and the frequencies of the genotypes, the translation landscapes of the VP3 and VP1 mutant swarms were built at conditions of 0.00 µg/ml (fig. 4*A* and *D*), 0.05 µg/ml (fig. 4*B* and *E*), and 0.20 µg/ml of AMD (fig. 4*C* and *F*). At the first glance, in conditions of no shutoff and moderate shutoff there were less diversity of phenotypes, that is, relative FLuc/RLuc ratio, in the VP1 fragment than in VP3, while in conditions of high cellular shutoff there were more diversity in VP1. Specifically, in VP3 the seven genotypes translated into 4, 3, and 1 phenotypes, in conditions of no, moderate, or high shutoff, respectively, while in VP1 the 12 genotypes translated into 1, 2, and 7 phenotypes.

The low phenotype diversity of VP1 in conditions of no shutoff, is likely due to the presence of many slowly translating codons (51% in VP1 vs. 44% in VP3; table 2), which may normalize the speed of translation between genotypes at a slow pace. In contrast, the low phenotype diversity of VP3 in conditions of high shutoff, in which the most common codons are rapidly translated, is likely associated to its lower proportion of slowly translated codons (15% in VP3 vs. 23% in VP1; table 2), which tend to balance the speed of translation between genotypes at a very rapid pace.

### Translation Efficiency G → P Maps: Codon Composition Influences HAV Evolvability

**Fig. 5.**
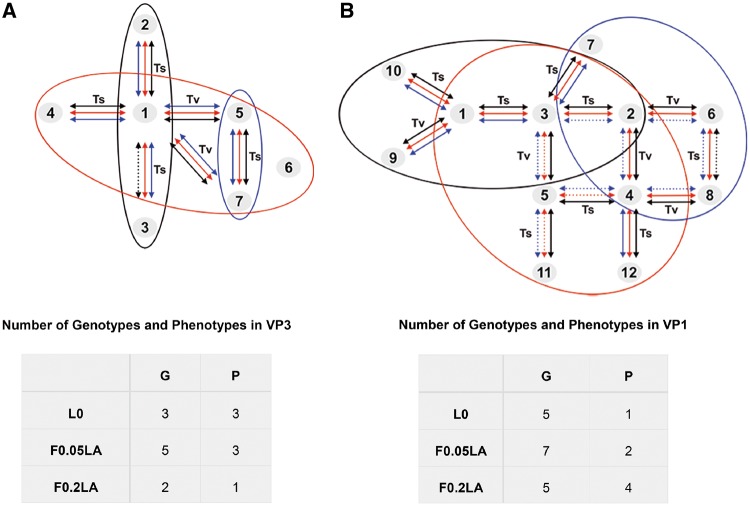
—Estimation of the translation efficiency robustness through G → P maps. The number of different genotypes and phenotypes for each fragment and population, and their location in the G → P maps of the VP3 (*A*) and VP1 (*B*) fragments is depicted. L0 population: black circles. F0.05LA population: red circles. F0.2LA population: blue circles. Arrows connect one mutation away genotypes. Continuous arrows connect genotypes expressing the same phenotype. Discontinuous arrows connect genotypes expressing different phenotypes. Black, red, and blue arrows denote absence, moderate, and high cellular shutoff conditions, respectively. *T*_s_ and *T*_v_ are transition and transversion mutations, respectively.

Obviously, not all potential genotypes resulting from one mutation step away surrounding each of the focal initial genotypes were found either in the VP3 or in the VP1 sequence space (fig. 2 and supplementary tables S1 and S2, Supplementary Material online). Considering only the initial dominant genotypes, VP3-1 and VP1-1, and excluding the nonsense mutations, 1,064 total genotypes and 228 synonymous genotypes and 1,185 total genotypes and 280 synonymous genotypes are expected in VP3 and VP1, respectively. On the contrary, only 3 and 5 one mutation away genotypes were found, respectively, indicating the huge constraints shaping the sequence space (figs. 2 and 5*A* and *B*).

Globally, 7 and 12 genotypes were detected in VP3 and VP1, respectively, which could be derived by successive one mutation away movements, but each of these genotypes did not give rise to a distinct phenotype (figs. 4 and 5*A* and *B*). In conditions of no shutoff the genotype:phenotype (G:P) ratio in VP3 was 7:4, in moderate shutoff 7:3, and in conditions of high shutoff 7:1. In contrast, in VP1 the same adaptation induced an overall increase of genotypes from 5 to 12 (fig. 5B). In this case, the G:P ratios were 12:1, 12:2, and 12:7 in conditions of no, moderate, and high shutoff, respectively. Looking at each particular population in their optimal conditions, the G:P ratios were of 3:3, 5:3, and 2:1 in VP3 and of 5:1, 7:2, and 5:4 in VP1 for L0, F0.05LA, and F0.2LA populations, respectively (fig. 5*A* and *B*).

**Fig. 6.**
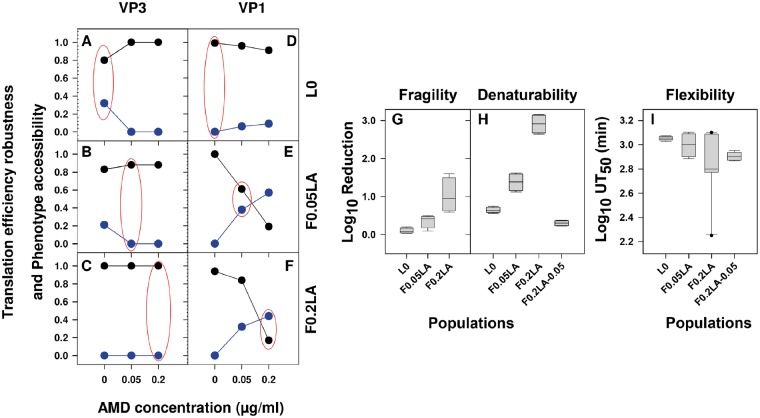
—Translation efficiency robustness and phenotype accessibility, and its relationship with folding diversity. (*A*–*F*) Translation efficiency robustness (black plots) and phenotype accessibility (blue plots). Analyses of VP3 (*A*, *B*, and *C*) and VP1 (*D*, *E*, and *F*) fragments of populations L0 (*A* and *D*), F0.05LA (*B* and *E*), and F0.2LA (*C* and *F*) under conditions of no (0.0 µg/ml of AMD), moderate (0.05 µg/ml of AMD), and high cellular shutoff (0.2 µg/ml of AMD) are shown. Red circles represent the optimum conditions of each population. (*G*–*I*) Capsid behavior under extreme conditions of populations L0, F0.05LA, and F0.2LA grown in their optimum conditions and population F0.2LA grown in conditions of moderate cellular shutoff (F0.2LA-0.05). Boxplot diagrams show the fragility to 300 MPa (high hydrostatic pressure) for 1 min (*G*), the denaturability at pH 2 for 1 h (*H*), and the plasticity for RNA uncoating measured as the log_10_ 50% uncoating time in minutes (*I*). The bottom and upper limits of the boxes represent the 25th and 75th percentiles, respectively. The bottom and upper whiskers represent the 5th and 95th percentiles, respectively. A solid black line represents the median.

The translation efficiency robustness and the phenotype accessibility for each of the three populations, in each of the three shutoff conditions, and for each fragment were estimated using the G → P maps (fig. 5*A* and *B*) and taking into consideration the frequency of each genotype (fig. 2 and supplementary fig. S2, Supplementary Material online). No differences were detected between passages 5 and 103 of L0 population (fig. 6*A* and *D* and supplementary fig. S5, Supplementary Material online), and being the former the real ancestor was used for the comparative analysis with F0.05LA and F0.2LA populations. Translation efficiency robustness in the VP3 region in population L0 progressively and significantly (*P* < 0.05) increased with shutoff from 0.80 to 1 (fig. 6A). In contrast, no significant variation was observed in populations F0.05LA (fig. 6B) and F0.2LA (fig. 6C), which showed a largely high robustness in all conditions, and in consequence their phenotype accessibility was overall very low. In contrast, in the VP1 fragment, the robustness of translation progressively and significantly (*P* < 0.05) decreased with shutoff in population L0 from 1 to 0.91 (fig. 6D), and particularly in populations F0.05LA from 1 to 0.19 (fig. 6E) and F0.2LA from 0.94 to 0.17 (fig. 6F). Accordingly, phenotype accessibilities increased. Overall, the robustness in the optimal conditions for each population were significantly (*P* < 0.05) different and of 0.80, 0.88, and 1.00 in VP3 and 1.00, 0.61, and 0.17 in VP1 for L0, F0.05LA, and F0.2LA, respectively. However, what it is more relevant is the robustness in the conditions to which populations had to be adapted, which may be an indication of the evolvability. The VP3 fragment showed a very low evolvability to conditions of shutoff, since the robustness of population L0 in conditions of moderate shutoff was of 1 and that of population F0.05LA in conditions of high shutoff was of 0.88 (fig. 6A–*C*). In contrast, the VP1 fragment showed higher evolvability, with a robustness of population L0 in conditions of moderate shutoff of 0.96 and of population F0.05LA in conditions of high shutoff of 0.19 (fig. 6D–*F*). In fact, the G → P maps indicated that while in VP1 adaptation to increasing shutoff conditions involved population expansions (fig. 5*B*), in VP3 this population spreading was only relevant during the first phase of adaptation and even there was a narrowing in conditions of high shutoff (fig. 5A). These different behaviors may be explained by the different codon composition of each fragment. About 28% of the codons of the VP1 fragment, correspond to the most common codon for each amino acid, in terms of human codon usage, and 23% to the least common codons; in contrast these figures in the VP3 fragment are 19% and 15%, respectively (table 2). The most and least common codons are usually considered to be rapidly and slowly translated, respectively; but the theoretical translation elongation rates of these two groups are not significantly different (supplementary fig. S6*A*, Supplementary Material online). Yet, analyzing separately the codons with an *R*_c_ below and over the median of each group, we found interesting results. While no differences between the groups were found analyzing the codons with an *R*_c_ below the median (supplementary fig. S6*B*, Supplementary Material online), the codons with an *R*_c_ over the median definitely did show significant differences (supplementary fig. S6*C*, Supplementary Material online). The most common group has a significantly higher *R*_c_ value than the least common (*P* = 0.02) and intermediate (*P* = 0.01) groups. Indeed, the codon frequency plays a role in controlling the actual translation rate, but in a shutoff-depending manner (fig. 3). In the absence of cellular shutoff, the most common codons are not as efficiently translated as in conditions of shutoff, and even in some cases may contribute to slow down the translation (fig. 3). The most common codons may become slowly translated codons by competition, due to the scarcity of their tRNAs mostly consumed in the translation of cellular mRNAs, and as such they may modulate the rate of translation in combination with the least common codons. Therefore, the occurrence of these two groups of codons may predict the evolvability to conditions of shutoff.

### Translation Efficiency Robustness versus Capsid Behavior Robustness

Since the codon composition has a direct impact on the speed of translation and in the robustness of the mutant swarm, it may in turn influence the diversity of capsid folds.

The translation efficiency robustness was significantly lower (*P* < 0.05) in VP3 than in VP1 in population L0 in the absence of shutoff (fig. 6*A* and *D*). However, the occurrence of a dominant genotype of VP3 (fig. 2), would contribute to the existence of a major translation phenotype. Similarly, in VP1, despite the occurrence of genotypes at frequencies ∼10% additionally to the dominant one (fig. 2), all showed the same translation phenotype (supplementary table S7, Supplementary Material online). Thus, in conditions of no shutoff, both capsid regions would end up into a single dominant phenotype, even displaying different codon compositions. Accordingly, in conditions of no shutoff, a dominant capsid fold could be expected for the L0 population. Capsid folding robustness was examined through the analysis of the diversity of several capsid features on box-and-whisker plots, including fragility to high hydrostatic pressure (HHP; 300 MPa for 1 min), acid denaturability (pH 2 for 1 h), and plasticity for RNA uncoating (50% uncoating time in minutes). Certainly, L0 population did show a very robust behavior (low diversity) in any of the three conditions tested, as shown by the very short boxes (fig. 6*G*–*I*), suggesting the occurrence of a single phenotype.

In conditions of moderate shutoff, the F0.05LA population in VP3 showed three translation phenotypes, the same number than L0 in its optimal condition of no shutoff (fig. 5 and supplementary table S6, Supplementary Material online). In contrast, in VP1 the F0.05LA population was less robust (fig. 6D and *E*) and showed two translation phenotypes instead of the single one of L0 (supplementary table S7, Supplementary Material online). This increase correlated with an expanded diversity of capsid behaviors in F0.05LA. Longer boxes and whiskers were observed in the F0.05LA population compared with the L0 population (fig. 6*G*–*I*), being significantly different regarding denaturability (*P* = 0.01) and flexibility (*P* < 0.00001).

Finally, in conditions of high shutoff, the translation efficiency of the VP3 fragment in population F0.02LA was highly robust (fig. 6C) and showed a very restricted phenotypic diversity (supplementary table S6, Supplementary Material online). In contrast, the VP1 translation robustness was very low (fig. 6*D*–*F*), and hence the number of phenotypes was quite remarkable with a clear increase compared with both L0 and F0.05LA populations in their optimal conditions (supplementary table S7, Supplementary Material online). The phenotypic diversity of VP1 translation efficiency correlated with a high diverse capsid performance in relation to fragility, denaturability, and flexibility (fig. 6*G*, *H*, and *I*). F0.2LA population showed significantly longer boxes and whiskers than L0 and F0.05LA populations, respectively, in relation to fragility (*P* = 0.003 and *P* = 0.003), denaturability (*P* < 0.00001 and *P* < 0.00001) and flexibility (*P* < 0.00001 and *P* < 0.00001).

The good correlation between the translation efficiency robustness of the VP1 fragment and the capsid folding robustness is likely related to the occurrence of a high proportion of codons from the most common group (table 2). These codons showed unexpected slow translation in conditions of no shutoff (fig. 3), and the need to maintain this slow rate would drive the adaptation to conditions of shutoff by increasing the genotype and phenotype diversity through mutations slowing down their efficiency of translation (table 4). Obviously, the capsid properties analyzed depend on the whole particle rather than on a piece of it, but the results suggest that translation of regions rich in codons of the most common type may be more influential in determining capsid folding than translation of regions poorer in this type of codons.

However, many of the detected mutations in the capsid region during the process of adaptation to cellular shutoff were nonsynonymous and consequently it can be argued that these amino acid replacements are indeed the reason for the occurrence of different phenotypes. To test this possibility, we changed the growing conditions of population F0.2LA that was then grown in conditions of moderate shutoff instead of high shutoff (F0.02LA-0.05), and we tested again the acid denaturability and the uncoating efficiency. Remarkably, population F0.2LA grown in conditions of moderate shutoff showed a more homogenous behavior (single phenotype) with significantly shorter boxes (*P* < 0.00001 and *P* < 0.00001 for denaturability and flexibility, respectively) than in conditions of high shutoff (fig. 6*H* and *I*). These results prove that capsid folding changes were environmental-dependent and that the amino acid replacements detected during the process of adaptation to shutoff could contribute but were not the main underlying reason of the capsid folding changes. Instead, a role of the translation efficiency and its environmental-dependent robustness is envisaged as a main contributor to capsid folding.

### Codon Usage Evolutionary Perspective

It has been suggested that viruses infecting humans show strong resemblance in terms of codon preferences to most mammalian hosts (Bahir et al. 2009); however, this is not the case for HAV, which shows very different preferences even compared with its current host.

In order to gain insight into this issue, a codon usage-based distance matrix was built between a range of eukaryotic organisms from protozoa to animals including, a collection of platyhelminthes, annelids, nematodes, molluscs, insects, birds, and mammals, and a range of viruses including HAV and several picorna-like viruses infecting the range of hosts (supplementary fig. S7, Supplementary Material online). Interestingly, the closest viruses to HAV were *Cripavirus* (CrPV), *Cryptosporidium* virus (CSpV), and Drosophila C virus (DCV). The closest eukaryotes were *Cryptosporidium parvum* (protozoa) followed at a certain distance by *Schistosoma mansoni* (platyhelminth) and *Brugia malayi* (nematode).

## Discussion

Viruses infecting both prokaryotes (Carbone 2008) and eukaryotes (Bahir et al. 2009) show strong codon adaptation to their hosts. Even, it has been recently proven that evolution shapes the codon content of the bacteriophage lambda early/late genes to fit them to the dynamic intracellular environment, that is, the *Escherichia coli* tRNA pools (Goz et al. 2017). Similarly, as tRNA pools may vary within a tissue depending on the differentiation status, it has been proposed that the match between the tRNA availability and codon usage may be a determinant of the papillomavirus late genes restricted expression to mature epithelium cells (Zhou et al. 1999). However, HAV is an exception to this rule and shows a deviated codon usage with respect its host. In an attempt to understand the underlying reasons of this deviated codon usage, we decided to perform experimental evolution under changing conditions of host tRNA demand. Artificially induced cellular transcription shutoff is a strong environmental perturbation for a virus adapted to conditions of no shutoff such as HAV. Consequently, it represents an ideal model for the study of the translation efficiency and its robustness using G → P maps, which in turn may be useful to infer HAV evolvability.

With this purpose, the effect of sequence space movements, occurring during the process of adaptation to cellular shutoff, on the capsid translation landscape was analyzed. Two powerful tools were used to build the G → P maps. Deep-sequencing was used to determine the type and frequency of genotypes, and a quantitative high-throughput screening assay using a bicistronic vector bearing the HAV IRES was used to calculate the translation efficiency of the detected genotypes. With this approach, we built maps for two different fragments of the HAV capsid coding region differing in their codon composition. These maps revealed that in L0 parental-type population, the high phenotype diversity observed in the VP3 fragment (low mutational robustness) is balanced with a very low genotype entropy, while in VP1, with the occurrence of a single phenotype (high mutational robustness), a higher genotype entropy is possible. This confirms that population diversity results from both the generation of and the tolerance to mutations (Lauring et al. 2013), which in HAV affect translation efficiency. Additionally, it indicates how constrained the HAV translation landscape is, which correlates with a highly robust capsid folding and in turn with a highly stable capsid.

While selection for translational robustness predicts a constraint on the nonsynonymous rate of evolution, selection for translational accuracy predicts a constraint on the synonymous rate of evolution (Wilke and Drummond 2006). Unexpectedly, while in VP1 *K*_a_ > *K*_s_ and in VP3 *K*_s_ ≫ *K*_a_, in L0 parental-type population, translation efficiency robustness was higher in VP1 than in VP3. However, these predictions do not account for the contribution of codon frequency in modulating the speed of translation elongation, and the results observed in the VP1 region of the parental-type virus may reflect the need to maintain the pace of translation, which is facilitated through nonsynonymous mutations. Actually, it has been described that the one-step nonsynonymous neighbors of a codon have similar tRNA abundances (Shah and Gilchrist 2010). Assuming a relationship between the codon frequency and the tRNA pools, the least and most common codons are expected to be slowly and rapidly translated, respectively (Tuller et al. 2010; Plotkin and Kudla 2011; Pechmann and Frydman 2013). However, HAV needs to confront an environment of unfair competition for the tRNAs, since is unable to induce the cellular shutoff (Ali et al. 2001), has a very inefficient IRES (Whetter et al. 1994) and has a deviated codon usage (Sánchez et al. 2003). In this scenario, the most common codons of the human genome, may behave as slowly translated codons by competition (Pintó et al. 2018). This interpretation correlates with the overrepresentation of mutations inducing a reduction of the *R*_c_ in the VP1 fragment (72% versus the randomly expected 46%) compared with VP3 (58% versus 55%), to balance the loss of the role of the slowly translated codons by competition during the adaptation to shutoff.

Assuming a critical role of the translation efficiency on the HAV capsid folding and in turn in its overall fitness, the VP1 G → P map can be used to estimate the evolvability to conditions of shutoff. The influence of mutational robustness on adaptation to environmental changes is controversial, with studies suggesting that robustness may impede adaptation (Ancel and Fontana 2000; Cowperthwaite et al. 2008; Parter et al. 2008) and studies suggesting just the contrary (Aldana et al. 2007; McBride et al. 2008). Draghi et al. (2010) have proposed a solution to this dilemma by mathematically proving that robustness (*q*) can accelerate adaptation if the number of phenotypes accessible to an individual by mutation (*K*) is smaller than the total number of phenotypes (*P*) in the fitness landscape. While adaptation is faster with an intermediate level of robustness, this optimal level of robustness increases as the ratio *K*/*P* decreases. The G → P map of the translation rate of the VP1 fragment of the L0 population revealed a robust (high *q*) neutral network of genotypes with *K*⋘*P*, and could adapt to conditions of shutoff only after quite a few number of passages (Aragonès et al. 2010). In contrast, the F0.2LA population showed a very low robustness (small *q*) but *K* < *P*. Consequently, the required number of passages for F0.2LA population to readapt to conditions of moderate shutoff were much lower than the number required for the adaptation of L0 (Aragonès et al. 2010). Additionally, although population F0.2LA evolved under changing conditions toward the edge of the neutral network of genotypes (Meyers et al. 2005), it shared some phenotypes with F0.05LA population, being able to “remember” the previous environment, which could facilitate its readaptation (Ruiz-Jarabo et al. 2000; Parter et al. 2008; Aragonès et al. 2010). On the contrary, L0 population evolved under constant conditions of shutoff toward a high robust neutral network of genotypes with very low phenotype accessibility (Meyers et al. 2005) and, thus, with no memory. In summary, L0 was less evolvable than F0.2LA and F0.05LA populations.

The lack of mechanisms to induce the cellular shutoff, as other picornaviruses do (Chase and Semler 2012; Walsh et al. 2013), combined with a deviated codon usage, suggests that HAV has coevolved with its host to efficiently compete for tRNAs, while ensuring a very robust capsid and an extremely low CpG content (de Oliveira Carneiro et al. 2018; Pintó et al. 2018). An alternative open question could be whether an ancestor with a codon usage optimized to an unknown host and with a mechanism to shut down its protein synthesis may have existed. While the first scenario is unusual among picornaviruses, the second is difficult to prove and it would involve a loss of the ability to shut down the cell protein synthesis. This latter point has been recently suggested through the study of the crystal structure of the 2B protein of HAV, which revealed an N-terminal fragment with striking structural similarities to the barrel domain of enteroviral 2 A proteases (Vives-Adriàn et al. 2015). Changes in substrate specificity of the 3 C protease of an ancestral virus would have resulted in the displacement of the 2 A/2B junction and the concomitant truncation and loss of function of the protease 2 A, disabling the virus to induce cellular shutoff (Vives-Adriàn et al. 2015). This latter situation, based on few mutations, might have had more chances for a successful host shift, than a complete adaptation of codons, in order to locally maintain the original translation efficiency and hence capsid folding robustness (Longdon et al. 2014). Likely, the ancestor virus would have also evolved toward a robust neutral network of translation genotypes with no memory to adapt rapidly to the new tRNA pools. It has recently been proposed that the avian influenza virus PB1 gene in H3N2 viruses may have evolved in humans by skewing codon usage to adapt to interferon-altered tRNA pools, and that this adaptation that took decades was driven first by genetic drift and by selection thereafter (Smith et al. 2018).

Since HAV is an exception to the general rule of human viruses of sharing codon preferences with most mammalian hosts (Bahir et al. 2009), a codon usage-based distance matrix was built to look for similarities with other related viruses and potential hosts. *Cripavirus* (CrPV), was the closest virus to HAV. Of note, based on a capsid structural phylogeny a distant relationship between HAV and CrPV has been recently described, and HAV has been suggested to be a link between “modern” picornaviruses and the more “primitive” precursor insect viruses (Wang et al. 2015). Several hepatoviruses have been recently isolated from small mammals and a Laurasiatherian host ancestor with an insectivorous diet has been suggested as a further link with a primordial insect-borne virus (Drexler et al. 2015). However, using codon usage distances the closest hosts belonged to protozoa, platyhelminthes, and nematodes. In contrast, insect hosts were far more distant to HAV. Hence, it is tempting to speculate that HAV may have evolved from an ancestor invading a more ancient host. It has been proposed that picorna-like viruses initially evolved in a Big Bang (Koonin et al. 2008) that antedated the radiation of the eukaryotes with viruses from the ancestral pool invading the emerging supergroups of eukaryotes (Koonin et al. 2008; Wolf et al. 2018). At this early stage, the virus–host coevolution was not a major issue, although it was common later (Koonin et al. 2008). However, regarding codon usage, unlike other picornaviruses HAV does not show such a coevolution, suggesting host shifts. Whatever was the case, an HAV ancestor evolved into the extant hepatoviruses, infecting humans, simians, marsupials, small mammals, sea mammals, reptiles, amphibians and ray-finned fishes (Anthony 2015; Drexler et al. 2015; de Oliveira Carneiro et al. 2018; Shi et al. 2018; Rasche et al. 2019).

## Supplementary Material

Supplementary data are available at *Genome Biology and Evolution* online.

## Supplementary Material

evz146_Supplementary_Data
