# Supplementary material for: The Critical Role of Codon Composition on the Translation Efficiency Robustness of the Hepatitis A Virus Capsid
Source: Genome Biol Evol. 2019 Jul 10;11(9):2439–56. doi: 10.1093/gbe/evz146 (PMC6735747; doi:10.1093/gbe/evz146)
Supplement: evz146_Supplementary_Data [file evz146_supplementary_data.docx]

**Table S1**. VP3 Genotypes.

| **Genotype** | **Codon replacement and frequency of use following the cell codon usage** | **VP3 position** | **Original R_c_**^1^ | **Mutated R_c_**^1^ |
| --- | --- | --- | --- | --- |
| **VP3-1** | - | - |  |  |
| **VP3-2** | CAT (H) (65%) 🡪 TAT (Y) (66%) | 50 | 2.86 | 11.37 |
| **VP3-3** | ATC (I) (100%) 🡪 ATT (I) (61%) | 46 | 4.02 | 11.09 |
| **VP3-4** | ATC (I) (100%) 🡪 ACC (T) (100%) | 46 | 4.02 | 1.61 |
| **VP3-5** | GCG (A) (25%) 🡪 GGG (G) (68%) | 12 | 13.39 | 8.38 |
| **VP3-6** | GCG (A) (25%) 🡪 GAG (E) (100%)  CAG (Q) (100%) 🡪 CGG (R) (92%)  GAA (E) (65%) 🡪 GAG (E) (100%)  AAA (K) (64%) 🡪 AAG (K) (100%)  ACT (T) (55%) 🡪 GCT (A) (67%)  TTT (F) (70%) 🡪 TTC (F) (100%)  TGT (C) (68%) 🡪 AGT (S) (50%) | 12  33  34  37  49  85  109 | 13.39  14.97  6.26  8.94  6.07  4.28  0.73 | 8.46  5.37  8.46  16.43  16.81  6.70  2.73 |
| **VP3-7** | GCG (A) (25%) 🡪 GAG (E) (100%) | 12 | 13.39 | 8.46 |

^1^Theoretical Rate of translation

**Table S2**. VP1 Genotypes.

| **Genotype** | **Codon replacement and frequency of use following the cell codon usage** | **VP1 position (aa)** | **Original R_c_**^1^ | **Mutated R_c_**^1^ |
| --- | --- | --- | --- | --- |
| **VP1-1** | - | - |  |  |
| **VP1-2** | ATC (I) (100 %) → GTC (V) (53 %)  ATT (I) (61 %) → GTT (V) (34 %) | 85  146 | 4.02  11.09 | 2.59  7.79 |
| **VP1-3** | ATT (I) (61 %) → GTT (V) (34 %) | 146 | 11.09 | 7.79 |
| **VP1-4** | ATC (I) (100 %) → GTC (V) (53 %)  TTG (L) (26 %) → TTC (F) (100 %)  ATT (I) (61 %) → GTT (V) (34 %) | 85  123  146 | 4.02  7.93  11.09 | 2.59  6.70  7.79 |
| **VP1-5** | TTG (L) (26 %) → TTC (F) (100 %)  ATT (I) (61 %) → GTT (V) (34 %) | 123  146 | 7.93  11.09 | 6.70  7.79 |
| **VP1-6** | ATC (I) (100 %) → GTC (V) (53 %)  ATT (I) (61 %) → GTT (V) (34 %)  GTA (V) (19 %) → GTT (V) (34 %) | 85  146  162 | 4.02  11.09  6.79 | 2.59  7.79  7.79 |
| **VP1-7** | TTC (F) (100%) → TTT (F) (70%)  ATT (I) (61 %) → GTT (V) (34 %) | 131  146 | 6.70  11.09 | 4.28  7.79 |
| **VP1-8** | ATC (I) (100 %) → GTC (V) (53 %)  TTG (L) (26 %) → TTC (F) (100 %)  ATT (I) (61 %) → GTT (V) (34 %)  GTA (V) (19 %) → GTT (V) (34 %) | 85  123  146  162 | 4.02  7.93  11.09  6.79 | 2.59  6.70  7.79  7.79 |
| **VP1-9** | AGC (S) (100 %) → ACC (T) (100 %) | 197 | 3.57 | 1.61 |
| **VP1-10** | ATT (I) (61 %) → ATC (I) (100 %) | 198 | 11.09 | 4.02 |
| **VP1-11** | TTG (L) (26 %) → TTC (F) (100 %)  ATT (I) (61 %) → GTT (V) (34 %)  GAC (D) (100 %) → GAT (D) (75 %) | 123  146  180 | 7.93  11.09  7.59 | 6.70  7.79  5.30 |
| **VP1-12** | ATC (I) (100 %) → GTC (V) (53 %)  TTG (L) (26 %) → TTC (F) (100 %)  ATT (I) (61 %) → GTT (V) (34 %)  GGT (G) (44 %) → AGT (S) (50 %) | 85  123  146  163 | 4.02  7.93  11.09  6.70 | 2.59  6.70  7.79  2.73 |

^1^Theoretical Rate of translation

**Fig. S1**. Time-kinetics of the translation rate of the VP1-1 fragment under the HAV IRES control, using the G1RCMsKp bicistronic vector, after transfecting FRhK-4 cells, and in the absence of AMD. The Firefly Luciferase (FLuc) activity is shown in red (left y-axis), and indicates the level of translation. The FLuc/RLuc ratio is shown in black (right y-axis), and is used as a quality control of the transfection and of the ratio between mRNA transcription and protein synthesis/degradation. An incubation time of 24h was chosen for the rest of the study.

**Fig. S2**. **Analyses of the mutant spectra of the L0 ancestor population at passage 103.** Genotype distribution of VP3 (left pie) and VP1 (right pie) is shown. The diversity, frequency of genotypes and the S_n_ values in the population L0 after 103 passages did not differ significantly from the L0 population at passage 5 (Figure 2 of main text) used throughout this work as the parental ancestor. One and 2 new genotypes emerged in VP3 (VP3-4) and VP1 (VP1-4 and VP1-5), respectively, as it happened in population F0.05LA. However, their frequencies in L0 at passage 103 were much lower than in population F0.05LA. Additionally, in VP1 one genotype (VP1-10) present at passage 5 was no further detected, while genotype VP1-9 increased its frequency.

**Table S3**. Influence of the type of mutations on the likelihood of codon frequency changes in a range of ± 10%.

|  | **Synonymous**  (25% of all mutations) | **Non-synonymous**  (75% of all mutations) |
| --- | --- | --- |
| **Codon frequency unchanged**  (37% of all mutations) | 13 % | 45 % |
| **Codon frequency changed**  (63% of all mutations) | 87 % | 55 % |

**Table S4.** **Analysis of the mutant spectra of the L0 population slightly mutagenized with 5-Fluorouracil (FU).** Genetic features include, the minimum and maximum nucleotide mutation frequency, the minimum and maximum non-synonymous mutation frequency, the rate of synonymous mutations per synonymous site (K_s_), the rate of non-synonymous mutations per non-synonymous site (K_a_) and the normalized Shannon entropy (S_n_).

|  | **VP3** | | **VP1** | |
| --- | --- | --- | --- | --- |
|  | **No drug** | **FU 80 µM** | **No drug** | **FU 80 µM** |
| **Minimum nucleotide mutation frequency** | 0 | 6.8 x 10^-4^ | 1.5 x 10^-4^ | 6.3 x 10^-4^ |
| **Maximum nucleotide mutation frequency** | 0 | 7.9 x 10^-4^ | 1.5 x 10^-4^ | 1.3 x 10^-3^ |
| **Minimum non-synonymous mutation frequency** | 0 | 5.4 x 10^-3^ | 1.5 x 10^-4^ | 9.6 x 10^-3^ |
| **Maximum non-synonymous mutation frequency** | 0 | 8.1 x 10^-3^ | 1.5 x 10^-4^ | 9.6 x 10^-3^ |
| **K_s_** | 0.0000 | 0.0146 | 0.0000 | 0.0028 |
| **K_a_** | 0.0000 | 0.0020 | 0.0027 | 0.0034 |
| **K_a_/K_s_** | - | 0.1385 | - | 1.070 |
| **Sn** | 0.00 | 0.25 | 0.08 | 0.34 |

**Table S5. Mutations detected** **during the process of adaptation to conditions of cellular shutoff, distributed by capsid genome fragments and type of codons, and their influence on the theoretic translation elongation rate (R_c_).** The types of codons were classified following the human codon usage in: the most common codon for each amino acid, the least common codon and other codons.

| **Mutations per type of codon and fragment^a^** | | | | | | | | | | | |
| --- | --- | --- | --- | --- | --- | --- | --- | --- | --- | --- | --- |
| **VP3** | | | | | | **VP1** | | | | | |
| **Most**  **common** | | **Least**  **common** | | **Other** | | **Most**  **common** | | **Least**  **common** | | **Other** | |
| 17 (19) | | 75 (15) | | 8 (66) | | 31 (28) | | 10 (23) | | 59 (49) | |
| **Mutations increasing or decreasing the R_c_**  **per type of codon and fragment^b^** | | | | | | | | | | | |
| **Most**  **common** | | **Least**  **common** | | **Other** | | **Most**  **common** | | **Least**  **common** | | **Other** | |
| **Δ↑** | **Δ↓** | **Δ↑** | **Δ↓** | **Δ↑** | **Δ↓** | **Δ↑** | **Δ↓** | **Δ↑** | **Δ↓** | **Δ↑** | **Δ↓** |
| 0 (34) | 100 (58) | 56 (30) | 44 (60) | 100 (42) | 0 (52) | 0 (40) | 100 (53) | 67 (56) | 33 (36) | 35 (43) | 65 (49) |

^a^Percentage of new mutations and mutations whose proportion increased compared to the dominant genotype in the mutant spectra of L0 population, distributed per codon classes. The percentage of expected mutations is shown in brackets. ^b^Influence of the mutations detected during the process of adaptation to cellular shutoff on the theoretical rate of translation elongation (R_c_): Δ↑ represents the percent of mutations increasing R_c_, and Δ↓ represents the percent of mutations decreasing R_c_. The percent of expected mutations is shown in brackets; the difference up to 100%, correspond to mutations with no influence on the R_c_.


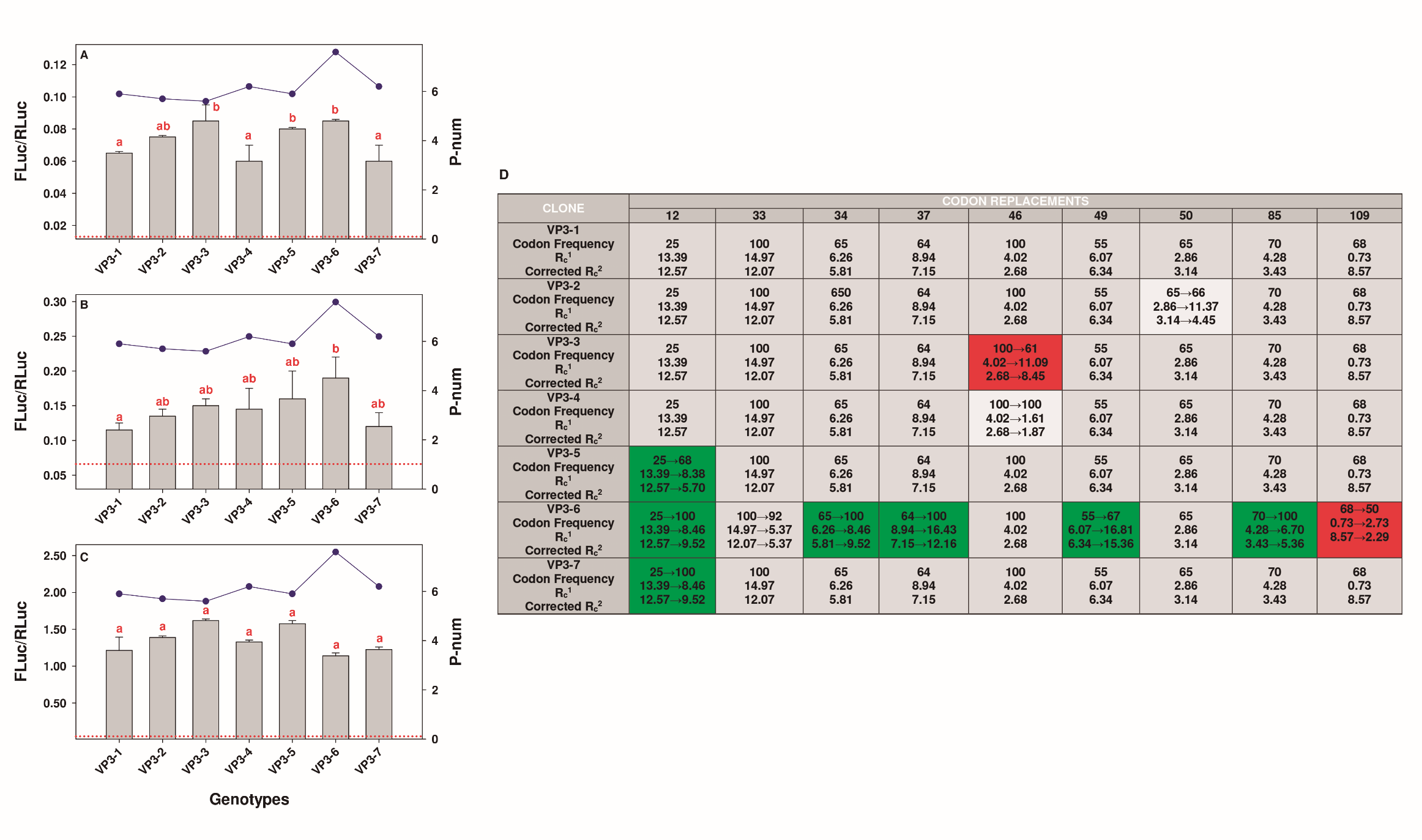


**Fig S3**. **Effect of mutations inducing changes in the codon frequencies in the translation efficiency**. Left panel: actual translation efficiency of the VP3 clones. Results are based on three different experiments, each including two replicas. (A) conditions of no shutoff, (B) conditions of moderate shutoff and (C) conditions of high shutoff. The dotted red line correspond to the average of a negative control corresponding to cells transfected with the digested vector alone. Statistically significant differences (p<0.05) are depicted by different combinations of letters (ab=a, ab=b; a≠b). The blue line plots represent the P-num, which predicts the potential occurrence of secondary structures in the RNA of the different genotypes; the lower the P-num value the higher the secondary structures in the RNA. Right panel: information on the codon replacements present in each clone, compared to the most abundant genotype (VP3-1) is indicated by colours. Red and green background colours represent a change to a less and more frequent codon, respectively, and light grey a mutation not changing the frequency.

^1^Theoretical translation elongation Rate (R_c_) of codons calculated using the data on human tRNA copy numbers available in <http://gtrnadb.ucsc.edu/Hsapi19/Hsapi19-summary.html>.

^2^R_c_ values corrected based on tRNA copy numbers available in (Iben and Maraia 2014).

**Fig S4. Translation efficiency of different VP1 and VP3 clones under the control of a mutated more active HAV IRES**. VP1 clones in conditions of high shutoff (A) and VP3 clones in conditions of no shutoff (B). The shutoff conditions were chosen based on the maximum translation diversity observed using the standard HAV IRES (Figure 3). The mutated IRES has higher activity (Pérez-Rodríguez et al 2016). Results are based on three different experiments, each including two replicas. The dotted red line correspond to the average of a negative control corresponding to cells transfected with the digested vector alone. Statistically significant differences (p<0.05) are depicted by different combinations of letters (ab=a, ab=b, a≠b; ae=a, ae=e, a≠e, etc.). No changes on the translation pattern were observed using the active IRES although the global FLuc/RLuc ratios were significantly higher (p < 0.003).

**Table S6.** Translation phenotypes of VP3 in each population and shutoff conditions.

| **Population**  **(condition)** | **Percent of genotypes**  **(genotypes)** | **Relative FLuc/RLuc activity**  **(phenotypes)** |
| --- | --- | --- |
| **L0 (0.00 µg/ml AMD)** | 99.00 (1) | 1.00 ± 0.12^bc^ |
|  | 3.26 (2) | 1.14 ± 0.18^ab^ |
|  | 0.74 (3) | 1.31 ± 0.24^a^ |
| **F0.05LA (0.00 µg/ml AMD)** | 40.10 (4) | 0.91 ± 0.06^c^ |
|  | 23.51 (1) | 1.00 ± 0.12^bc^ |
|  | 17.95 (5) | 1.21 ± 0.14^ab^ |
|  | 12.41 (6) | 1.29 ± 0.15^a^ |
|  | 6.03 (7) | 0.96 ± 0.09^bc^ |
| **F0.2LA (0.00 µg/ml AMD)** | 99.35 (7) | 0.96 ± 0.09^bc^ |
|  | 0.65 (5) | 1.21 ± 0.14^ab^ |
| **L0 (0.05 µg/ml AMD)** | 100 (1, 2, 3) | 2.04 ± 0.35^a^ |
| **F0.05LA (0.05 µg/ml AMD)** | 58.05 (4, 5) | 2.35 ± 0.68^ab^ |
|  | 29.54 (1, 7) | 1.83 ± 0.28^b^ |
|  | 12.41 (6) | 2.93 ± 0.56^a^ |
| **F0.2LA (0.05 µg/ml AMD)** | 100 (5,7) | 2.17 ± 0.60^b^ |
| **L0 (0.2 µg/ml AMD)** | 100 (1, 2, 3) | 21.46 ± 5.64^a^ |
| **F0.05LA (0.2 µg/ml AMD)** | 100 (1, 4, 5, 6, 7) | 20.16 ± 6.18^a^ |
| **F0.2LA (0.2 µg/ml AMD)** | 100 (5, 7) | 21.82 ± 7.97^a^ |

Statistically significant differences (p < 0.05) of the FLuc/RLuc activity, relative to the FLuc/RLuc of the VP3-1 clone in 0.00 µg/ml AMD, in each shutoff condition are depicted by different combinations of letters. However, since a=ab, b=ab but a≠b, etc., each combination of letters represents a distinct phenotype.

**Table S7.** Translation phenotypes of VP1 in each population and shutoff conditions.

| **Population**  **(condition)** | **Percent of genotypes**  **(genotypes)** | **Relative FLuc/RLuc activity**  **(phenotypes)** |
| --- | --- | --- |
| **L0 (0.00 µg/ml AMD)** | 100 (1, 2, 3, 9, 10) | 1.01 ± 0.20^a^ |
| **F0.05LA (0.00 µg/ml AMD)** | 100 (1, 2, 3, 4, 5, 11, 12) | 1.01 ± 0.19^a^ |
| **F0.2LA (0.00 µg/ml AMD)** | 100 (2, 4, 6, 7, 8) | 1.01 ± 0.19^a^ |
| **L0 (0.05 µg/ml AMD)** | 100 (1, 2, 3, 9, 10) | 1.65 ± 0.41^a^ |
| **F0.05LA (0.05 µg/ml AMD)** | 73.65 (1, 2, 3, 4, 11, 12) | 1.62 ± 0.42^a^ |
|  | 26.35 (5) | 4.48 ± 3.17^b^ |
| **F0.2LA (0.05 µg/ml AMD)** | 100 (2, 4, 6, 7, 8) | 1.64 ± 0.56^a^ |
| **L0 (0.2 µg/ml AMD)** | 81.54 (1, 9) | 9.92 ± 3.03^bcd^ |
|  | 11.52 (3) | 8.36 ± 1.85^cd^ |
|  | 3.53 (2) | 2.39 ± 1.51^e^ |
|  | 1.93 (10) | 11.18 ± 3.32^bc^ |
| **F0.05LA (0.2 µg/ml AMD)** | 37.94 (1, 4) | 9.81 ± 2.75^bcd^ |
|  | 26.35 (5) | 21.12 ± 4.75^a^ |
|  | 22.07 (2) | 2.39 ± 1.51^e^ |
|  | 12.24 (3) | 8.36 ± 1.85^cd^ |
|  | 1.40 (11, 12) | 5.72 ± 1.44^de^ |
| **F0.2LA (0.2 µg/ml AMD)** | 63.57 (4) | 10.23 ± 2.69^bcd^ |
|  | 24.27 (2) | 2.39 ± 1.51^e^ |
|  | 8.18 (7, 8) | 6.98 ± 1.51^de^ |
|  | 3.99 (6) | 13.82 ± 4.82^b^ |

Statistically significant differences (p < 0.05) of the FLuc/RLuc activity, relative to the FLuc/RLuc of the VP1-1 clone in 0.00 µg/ml AMD, in each shutoff condition are depicted by different combinations of letters. However, since a=ab, b=ab but a≠b, etc., each combination of letters represents a distinct phenotype.

**Fig. S5**. Translation efficiency robustness (black plots) and phenotype accessibility (blue plots) of the VP3 and VP1 fragments of the L0 population at passage 103, under conditions of no (0.0 µg/ml of AMD), moderate (0.05 µg/ml of AMD) and high cellular shutoff (0.2 µg/ml of AMD). No differences with respect passage 5 are observed (Figure 6 panels A and D.)

**Fig. S6**. **Theoretical rate of translation of the most common, the least common and the other codons for each amino acid.**  **(A)** Box-and-whisker plots considering all codons belonging to each group. The bottom and top whiskers represent the values including 10% and 90%, respectively, of the sample values; the bottom and top of the box represent the first and third quartiles, respectively, and the middle solid line represents the second quartile. The discontinuous line represents the mean and the points correspond to outlier values. No significant differences were found between the R_c_ of each group of codons. However, analysing separately the codons below **(B)** and above **(C)** the median, we could observe that while the codons below the median do not behave differently depending on their abundance, the codons above the median did show differences. Indeed, the mean of the most common codons was significantly higher than the mean of the least common (p = 0.02) and other (p = 0.01) groups (a≠b).

**
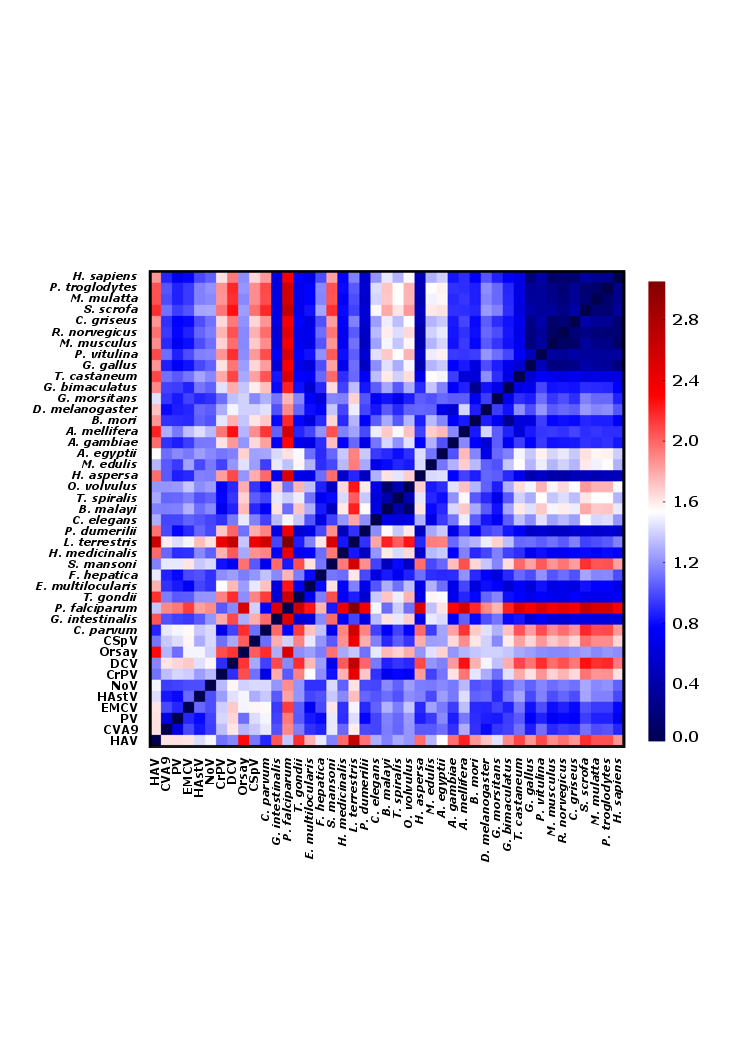
**

Picornaviruses: *Hepatovirus* (HAV), *Enterovirus B* (CV-A9), *Enterovirus C* (PV) and *Cardiovirus* A (EMCV). Picorna-like viruses: *Mammastrovirus* (HAstV), *Norovirus* (NoV) and *Cripavirus* (CrPV and DCV), a member of the *Nodaviridae* family infecting *Caenorhabditis elegans* (Orsay), a member of the *Partitiviridae* family infecting *Criptosporidium parvum* (*Cryspovirus*; CSpV). Protozoa: *Cryptosporidium parvum*, *Giardia intestinalis*, *Plasmodium falciparum* and *Toxoplasma gondii*. Platyhelminthes: *Echinococcus multilocularis*, *Fasciola hepatica* and *Schistosoma mansoni*. Annelids *Hirudo medicinalis*, *Lumbricus terrestris*, *Platynereis dumerilii*. Nematodes: *Caenorhabditis elegans*, *Brugia malayi*, *Trichinella spiralis* and *Onchocerca volvulus*. Molluscs *Helix aspersa*, and *Mytilus edulis*. Insects: *Aedes aegyptii*, *Anopheles gambiae*, *Apis mellifera*, *Bombyx mori, Drosophila melanogaster*, *Glossina morsitans*, *Gryllus bimaculatus*, and *Tribolium castaneum*. Aves: *Gallus gallus*. Mammals: *Phoca vitulina*, *Mus musculus*, Rattus norvegicus, *Cricetulus griseus*, *Sus scrofa*, *Macaca mulatta*, *Pan troglodytes* and *Homo sapiens*.

**Fig. S7. Distance matrix for the similarity in codon usage between a series of viruses and hosts.** A codon usage-based Euclidean distance matrix between several picornaviruses and picorna-like viruses and a range of eukaryotic organisms from protozoa to animals including platyhelminthes, annelids, nematodes, molluscs, insects, aves and mammals was built using the DendroUPGMA program (<http://genomes.urv.cat/UPGMA/>). The codon usage of all these organisms was available at the Codon Usage Database (<http://www.kazusa.or.jp/codon/>), and was based in a significant number of sequenced genes. The obtained matrix was used to build a heat map using the Python library Matplotlib. Codon usage distance is represented by a gradient of colors from dark blue (maximum similarity) to dark red (minimum similarity).
